# Enterectomy—End to End Anastomosis—Cholecyst-Enterostomy—With Murphy Button

**Published:** 1894-01

**Authors:** W. B. Rogers

**Affiliations:** Memphis, Tenn.; Professor of Principles and Practice of Surgery and Clinical Surgery at Memphis Hospital Medical College


### ENTERECTOMY—END TO END ANASTOMOSIS—CHOLECYST-ENTEROSTOMY—WITH MURPHY BUTTON.*

By W. B. ROGERS, M. D.,
Professor of Principles and Practice of Surgery and Clinical Surgery at Memphis Hospital Medical College.
MEMPHIS, TENN.

CASE 1ST.—*Resection of Small Intestine for Gangrene, End to End Anastomosis with Murphy Button.*

Thursday, October 12th, 1893, Robert Hannon, male, twenty years of age, jumped from a wagon to the ground. The jar caused the descent of a right inguinal hernia to which he had been subject for several years. He had theretofore been able to reduce the hernia, whenever it appeared, without the aid of a physician. On this occasion, however, he failed, and called his physician, who without the aid of an anesthetic, and with very little effort by taxis, succeeded in pressing the hernia apparently back into the abdomen. The patient, however, continued to complain of pain extending from the inguinal canal towards the umbilicus. There was a slight fullness or induration at the site of the internal ring, but apparently within the abdominal cavity. The patient's general condition was nearly normal. During the next two days calomel, salts and oil, failed to act on the bowels. So also did repeated enemas of large quantities of water fail to bring away fecal matter. The abdomen was gradually becoming distended and tympanitic, and when I saw him two days after the occurrence of the hernia, I found a belly greatly swollen, tympanitic, with marked tenderness from the umbilicus down to the inguinal canal on the right side. He was vomiting a dirty, greenish looking fluid with distinctly fecal odor.

*Read before the Southern Surgical and Gynecological Association, New Orleans' November 15th, 1893.

Pulse and temperature about normal. I invaginated the scrotum with my finger, carrying it well up into the inguinal canal, but was unable to feel the hernia there, though just above Poupart's ligament, and just under the internal ring, there remained a small induration, indistinctly felt by reason of distention of the abdomen. The conclusion was that the hernia had either been reduced in its sac, or else that both had been well pressed up the inguinal canal, and between the peritoneum and wall proper. An operation was at once decided upon. An incision three inches long was made over the site of the appendix. At least a pint of blood-stained water escaped from the peritoneal cavity. Inserting my two fingers I detected a constriction at the internal ring, and succeeded in drawing out eight inches of intestines. This I brought through the abdominal wound to the outer surface of the abdomen. It was so congested that at each end of the eight inches, where the encircling band had constricted it, the peritoneum had given away and gangrene was imminent. I immediately cut out ten inches of intestine and made an end to end approximation by means of Murphy's button. All hemorrhage was arrested, the abdomen cleansed, and drainage tube so placed as to reach the bottom of the pelvis. The wound was closed with silk suture; the patient taken off the table with a pulse of 80. The reaction that night was pronounced, temperature reaching 103°, but fell promptly by morning to 99½°. His pulse from that time on did not go above 86; his temperature ranging under 100°.

Fifteen grains of calomel were given the night after the operation; ten grains were repeated the next day, and by means of oft-repeated large enemas the intestinal canal was thoroughly emptied of all gas and fecal matter at the end of sixty hours. The patient's general condition was good all of the time after the operation, and he expressed himself ready at any time to get out of bed and go to work. Thirty-two days have now elapsed and his bowels are acting every day without medicine. His appetite is good and he is eating ordinary food. The drainage tube was removed on the third day. The button was passed at the end of the seventh day.

(The patient was then presented for examination by the Fellows of the Association.)

CASE 2D.—*Distended Gall Bladder, Cholecyst-enterostomy by Murphy's Button.*

Capt. Elliott; fifty-three years of age, of sedentary habits, and suffered more or less with dyspepsia for years. Six months before he became very much jaundiced. The liver was greatly enlarged; he had no fever; pulse feeble at sixty beats per minute; was emaciated to almost the last degree. There was great pain over the pyloric region, with tenderness all along the liver, which projected in a uniform line three inches and a half below the border of the ribs. At the anterior axillary line there was a distinct projection downwards from the liver reaching the anterior superior spinous process of the ilium. The case had been diagnosed by several physicians as cancer of the liver. While the symptoms pointed strongly to such a condition, I was not willing to consent to the diagnosis without an operation. This growth had been *so rapid;* had *overreached* the rapidity of growth so characteristic of malignance. I detected what I thought to be the rounded end of the gall bladder projecting at the lowest point of the enlarged liver. An incision was made two and a half inches below the anterior extremity of the ninth rib extending downwards three inches. Upon entering the abdomen I found the liver greatly enlarged, very much congested; it was blue but its surfaces were smooth and projecting; at the lowest point was the gall bladder. I succeeded in bringing the fundus of the gall bladder in the incision, and opening it evacuated fourteen ounces of bile. With a probe introduced into the gall bladder, and my two fingers in the abdominal cavity, I was enabled to follow the cystic to the common duct, but no further. I examined with my two fingers the under surfaces of the liver, and found no signs of malignant disease. I tested the common duct to the duodenum, and could find neither gall stone nor malignant growth, but the liver was so swollen that a stone in the common duct might easily have escaped touch.

The condition of the patient at this time admonished a rapid completion so that I did not attempt a cholecyst-enterostomy, but sutured the gall bladder to the anterior wall of the abdomen, hoping that under the use of liberal douches of hot water I might succeed in clearing out the common duct. He reacted slowly after the operation, but in a few days was out of all danger. The hot water douching was tried, and every effort made to pass a probe along the common duct without success.

At the end of three weeks he had gained some in strength, his appetite was good, and his color had nearly resumed the natural. The liver had receded beneath the ribs. In this state I allowed him to return to his home to recuperate before I operated for cholecyst-enterostomy.

Patient returned after three weeks with general condition very little improved, but the absence of bile from his bowel seemed to have prevented his gaining in flesh. The prospect of improvement in the absence of bile was so unpromising that, although his condition was bad, in consultation it was deemed advisable to connect the gall bladder and the bowel. At the time of this operation I again explored the track of the common duct and felt two distinct enlargements, believed to be calculi. The case was not one to waste time in efforts at removal of calculi from the common duct, so I hastily did the cholecyst-enterostomy and used the Murphy button. The case progressed favorably, the button coming away from the fistula in the abdomen on the seventh day. We had much trouble in keeping the food from escaping *via* the gall bladder. Most of the bile passed into the bowel. His condition for ten days was precarious indeed, but he slowly improved, and he went to his home at the end of four weeks. There he gained about fifteen pounds in flesh and was slowly but steadily improving until three months after the operation, when he was taken with diarrhea, soon becoming dysentery, and he died on the third day.

The operation, however, of cholecyst-enterostomy with the Murphy button, proved entirely satisfactory, and so far as an operation was concerned is to be regarded as a success in this case.

